# Numerical study of sediment scour at meander flume outlet of boxed culvert diversion work

**DOI:** 10.1371/journal.pone.0275347

**Published:** 2022-09-30

**Authors:** Hien T. T. Le, Chien Van Nguyen, Duc-Hau Le

**Affiliations:** 1 Faculty of Water Resources Engineering, Thuyloi University, Dong Da, Hanoi, Vietnam; 2 Hydraulic Construction Institute, Dong Da, Hanoi, Vietnam; 3 Faculty of Computer Science and Engineering, Thuyloi University, Dong Da, Hanoi, Vietnam; Tongji University, CHINA

## Abstract

**Background:**

Sediment scour at downstream of hydraulic structures is one of the main reasons threatening its stability. Several soil properties and initial input data have been studied to investigate its influence on scour hole geometry by both physical and numerical models. However, parameters of resistance affecting sedimentation and erosion phenomena have not been carried out in the literature. Besides, the auxiliary-like wing walls prevalently used in many real applications have been rarely addressed for their effect on morphological change.

**Results:**

In this study, a 3D Computational Fluid Dynamics model is utilized to calibrate the hydraulic characteristics of steady flow going through the culvert by comparison with experimental data, showing good agreement between water depth, velocity, and pressure profiles at the bottom of the boxed culvert. The results show that a grid cell of 0.015 m gave minimum NRMSE and MAE values in test cases. Another approach is numerical testing sediment scour at a meander flume outlet with a variety of roughness/*d*_*50*_ ratio (*c*_*s*_) and diversion wall types. The findings include the following: *c*_*s*_ = 2.5 indicates the close agreement between the numerical and analytical results of maximum scour depth after the culvert; the influence of four types of wing wall on the geometrical deformation including erosion at the concave bank and deposition at the convex bank of the meander flume outlet; and two short headwalls represent the best solution that accounts for small morphological changes.

**Conclusions:**

The influence of the roughness parameter of soil material and headwall types on sediment scour at the meander exit channel of hydraulic structure can be estimated by the numerical model.

## 1. Introduction

In dam projects, river diversion is considered the first step in the dam construction [1]. The issue is studied for three types of conveyance structures: i) tunnels and culverts, ii) open channels, and iii) overspill structures in the permanent works. Diversion works are temporary structures with short lifetimes, so their stability is often not a primary concern. However, water released into a river from tunnels or culverts may cause scouring of the riverbed, downstream of the cofferdam [1]. Therefore, the optimal design of the diversion system guarantees workshop safety and prevents possible damages resulting from conveying water during construction. The knowledge of anticipated local scours geometry has been the main concern of engineers or researchers for years because it is a significant criterion for the proper design of sluice outlet foundation [2–5]. Hence, predicting local scours after water conveyance structures such as spillways, outlet works, etc., has been widely studied to discover adequate protection solutions for the construction.

Numerous researchers have investigated the sediment scour problem. Physical models, artificial intelligence (AI) approaches, and numerical models have been efficient tools in this issue. Physical models are usually used to build empirical equations to calculate maximum scour depth and scour hole geometry [2,6–11]. However, physical models also exposed several limitations including time-consuming and costly. Especially, it is not flexible or easy to change the dimension or to install auxiliary work as well as the initial conditions, and boundary conditions during experimenting. Besides, the narrow range of physical conditions causes limitations when applying these empirical equations in case studies. On the other hand, some Artificial Intelligence (AI) approaches have been recently applied to predict the maximum scour depth in hydraulic structures [12,13]. A study by [14] showed that AI approaches were a suitable platform to reach the scour depth prediction with a permissible level of accuracy rather than empirical equations. However, to the best of our knowledge, AI approaches have not been proposed to predict the location of the maximum scour depth and other scour hole geometries [12]. In addition to the above approaches, numerical models are known to be more flexible, thus avoiding the limitations of physical models [11,15,16]. The numerical result also gives a comprehensive representation of hydraulic features of the whole computational domain. A number of well-known Computational Fluid Dynamics (CFD) models including OpenFoam, Ansys Fluent, Flow 3D, etc., have been widely utilized in this field [8,17–19]. These numerical models based on the coupling of the Volume Fluid Method and Navier Stokes equations have played important roles in simulating sediment scour issues due to the help of state-of-the-art 3D CFD models [10,20]. The robustness and effectiveness of commercial Flow 3D software are proven in practical applications [21,22]. In addition, Flow-3D was a useful software to simulate the local scour since it gives a significant association between the simulation and experiment [23,24]. However, roughness/*d*_*50*_ (*c*_*s*_) standing for the resistance of bed material in Flow 3D is not well studied in those studies although this parameter affects significantly the numerical solution of the local scour [8]. Additionally, headwalls at culvert outlets have been used prevalently in controlling outflow to direct in one main direction. The construction of headwalls against the flow causes a difference in velocity distribution that will generate different scouring mechanisms at downstream flumes. Previous works often study the effect of this device on flow regimes in exit channels (e.g., water depth and velocity distributions). However, there are only a few physical studies that addressed headwall design issues and local scour phenomena affected by headwalls [2,4,25]. The effect of wing walls and the effect of bed resistance parameters are underexplored in the previous numerical studies. Therefore, in this study, we intend to analyze the effect of the headwall on the geometry of scour hole after culvert as well as the erosion and deposition at the meander exit channel of diversion work using this numerical model. Besides, the hydraulic characteristics of meandering flumes are usually much more complex than those of straight flumes due to the occurrence of Coriolis force and whirlpool flow [3]. Therefore, three objectives are investigated: validate the presented model (Flow 3D) by comparing numerical results with observed data and analyze mesh sensitivity with three mesh resolutions; study the most reasonable parameter *c*_*s*_ in simulating maximum scour depth (*d*_*s max*_); discover the effects of wing wall types on the morphological pattern at the outlet of culvert and exit meander channel.

Some novel investigations can be listed: The numerical model Flow 3D is an efficient tool to simulate flow over culvert and bed deformation on exit meander channel. *c*_*s*_ = 2.5 is a reasonable parameter in evaluating *d*_*s max*_. Two long headwalls cause the deepest scour hole while two short walls create the smallest sand removal volume. The meandering of the exit channel induces erosion at the concave bank and deposition at the convex bank.

## 2. Material and methods

### 2.1. Physical model

**Fig 1:**
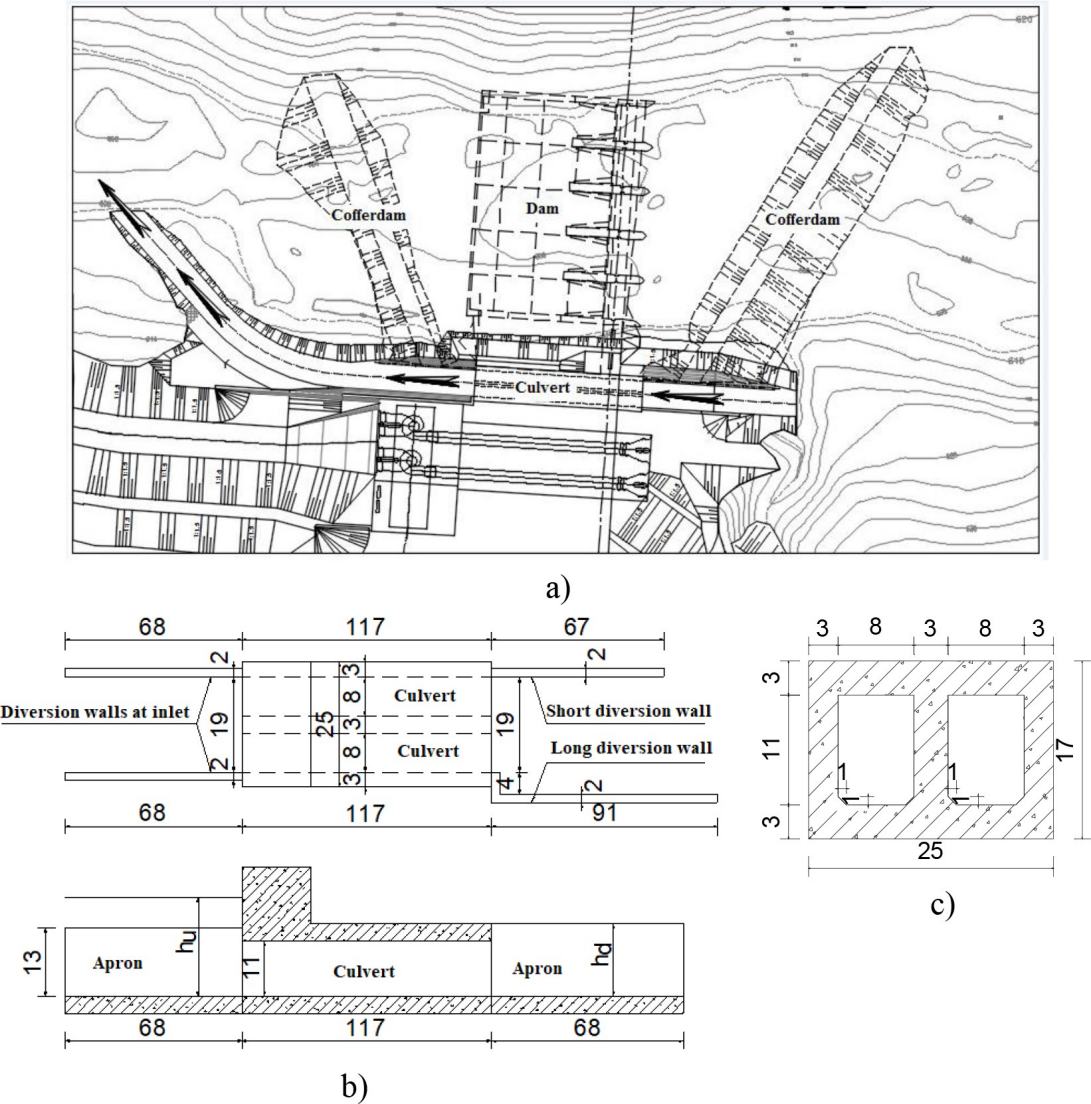
Schematic view of the physical model. a) Plan view of headwork; b) Plan view and longitudinal view of the two-box culvert; c) Cross-section of the two-box culvert (dimensions in cm).

Fig 1 shows a schematic view of the physical model of Dong Nai diversion work in the hydraulic laboratory of Thuyloi University, Vietnam with a ratio of 1/60 to examine its conveyance capacity. The diversion work including a two-box culvert, approach, and exit channels conveyed water from the upstream to the downstream of coffer dams. The intake was 117 cm in length, 11 cm in height, and 8 cm in width. Prismatic upstream and exit flumes were trapezoidal cross-sections with a 19 cm-bed width and 1.5 cm of side slope. Two wing walls at the entrance of the culvert were 68 cm in length, 13 cm in height, and 2 cm in width. Conversely, the long wall at the culvert installed on the concave bank was 91 cm in length, 13 cm in height, and 2 cm in width, while the short wall on the convex side had dimensions that were 67 cm in length, 5.0 cm in height, and 2 cm in width. The exit channel had a meander with a radius R = 125 cm [26].

The purpose of the physical model was to investigate the conveyance capacity of the boxed culvert in both dry and rainy seasons. In the flood season, water was conducted by both temporal intake and spillways, while in the dry season, water was drained out only by the culvert. In this study, three operating conditions incorporated with the dry season were selected (Table 1).

**Table 1:** Operating conditions.

Run	Discharge	Upstream water depth	Tail water depth
	*Q* (L/s)	*h*_*u*_ (m)	*h*_*d*_ (m)
1	8.32	0.120	0.059
2	13.95	0.182	0.069
3	16.14	0.194	0.070

In fact, the physical model did not carry out sediment issues on the exit channel because of the poorly measuring instruments in the laboratory. The measurement and observation were only done with pressure heads along the box tubes of the culvert, water level and velocity at upstream, and downstream sections, respectively [26]. In the second phase of this project, a mathematical model was required to assess hydraulic performance including velocity, vorticity, water depth profiles at cross sections, and sediment transport in the whole conveyance structure.

### 2.2. Governing equations

The commercial software package Flow 3D was built to solve Navier–Stokes equations by the Volume of Fluid (VOF) method, which is based on the conservation of two masses and momentums. Recently, this model has been increasingly used because it can be applied to simulate many sophisticated hydrodynamic problems.

Flow 3D provides two turbulent models to describe turbulent flow: Reynolds Averaged Navier Stokes (RANS) and Large Eddy Simulation (LES). The renormalization group (RNG), which is one of the RANS types, was selected due to its capacity to simulate flow over hydraulic works [27]. Moreover, the deformation of bed geometry can be demonstrated by the sediment scour module. Ten non-cohesive soil species, which are different in terms of mass density, main grain size, entrainment and transport coefficients, and critical shear stress can be added to the model as multilayers of bed information. This model can simulate the sediment transport process, which includes settling, packing, advection, bedload transport, entrainment, and depositions for each species, using the Flow 3D user manual [27].

#### 2.2.1.Continuity equation

The continuity equation is established by mass conservation. In general, bedload and suspended transport are used to describe the movement of sand particles in fluid flow. In the mathematical model, the bed boundary can be considered as a packed one if the local scour occurred at that place. The morphology of the packed boundary is estimated based on the conservation of mass. This process includes bedload transport, absorption, and deposition. The suspended sediment is estimated by sediment concentration and is considered a constraint at each computational cell. For each soil type, this term is estimated by the following continuity equation:

$$V_{f}\frac{\partial \rho }{\partial t}+\frac{\partial }{\partial x}(\rho uA_{x})+\frac{\partial }{\partial y}(\rho vA_{y})+\frac{\partial }{\partial z}(\rho wA_{z})=0$$ (1)

where *V*_*f*_ is volume fraction; *ρ* is fluid density; *u*, *v*, and *w* are velocity components in the *x*, *y*, and *z* directions, respectively; and *A*_*x*_, *A*_*y*_, and *A*_*z*_ are the area fractions.

#### 2.2.2. Momentum equations

Three momentum equations in the x, y, and z directions are as follows:

$$\begin{array}{l} \frac{\partial u}{\partial t}+\frac{1}{V_{F}}(uA_{x}\frac{\partial u}{\partial x}+vA_{y}\frac{\partial u}{\partial y}+wA_{x}\frac{\partial u}{\partial z})=-\frac{1}{\rho }\frac{\partial p}{\partial x}+G_{x}+f_{x}\\ \frac{\partial v}{\partial t}+\frac{1}{V_{F}}(uA_{x}\frac{\partial v}{\partial x}+vA_{y}\frac{\partial v}{\partial y}+wA_{x}\frac{\partial v}{\partial z})=-\frac{1}{\rho }\frac{\partial p}{\partial y}+G_{y}+f_{y}\\ \frac{\partial w}{\partial t}+\frac{1}{V_{F}}(uA_{x}\frac{\partial w}{\partial x}+vA_{y}\frac{\partial w}{\partial y}+wA_{x}\frac{\partial w}{\partial z})=-\frac{1}{\rho }\frac{\partial p}{\partial z}+G_{z}+f_{z} \end{array}$$ (2)

*G*_*x*_, *G*_*y*_, and *G*_*z*_ are the body accelerations, and *f*_*x*_, *f*_*y*_, and *f*_*z*_ are the viscous accelerations.

#### 2.2.3. Sediment scour model

The sediment transport process is often described in two phases, namely bedload transport and suspended transport. Bedload transport illustrates the motion of soil particles, such as rolling, hopping, and sliding along the packed bed surface due to the shear stress. Bedload transport means the movement of sand particles along the bed channel, regardless of whether some of them become suspended movement. The empirical formulas estimating bedload transport applied in Flow 3D were Meyer-Peter Muller, Nielsen, or Van Rijin [28,29].

The critical Shields parameter *θ*_*cr*_ is used to define the critical bed shear stress *τ*_*cr*_, at which sediment movement begins for both entrainment and bedload transport, which is applied to the horizontal bed.


$$\theta_{cr}=\frac{\tau_{cr}}{gd_{50}(\rho_{s}-\sigma )}$$ (3)

The Soulsby–Whitehouse equation is used to estimate the critical shear stress as follows:

$$\theta_{cr}=\frac{0.3}{1+1.2d_{*}}+0.055(1-e^{-0.02d_{*}})$$ (4)

$$d_{*}=d_{50}{\left[\frac{g(\rho_{s}/\rho -1)}{v}\right]}^{1/3}$$

where *ν* is the kinematic viscosity of the fluid, *ρ*_*s*_ is the soil mass density, and *ρ* is the fluid mass density.

The suspended sediment concentration is calculated by solving the following equation:

$$\frac{\partial C_{s}}{\partial t}+\nabla .(C_{s}.u_{s})=\nabla .\nabla (K.C_{s})$$ (5)

where *C*_*s*_ is the suspended sediment mass concentration, which is defined as the sediment mass per volume of fluid–sediment mixture; *K* is the diffusivity; and **u**_s_ is the sediment velocity.

**Fig 2:**
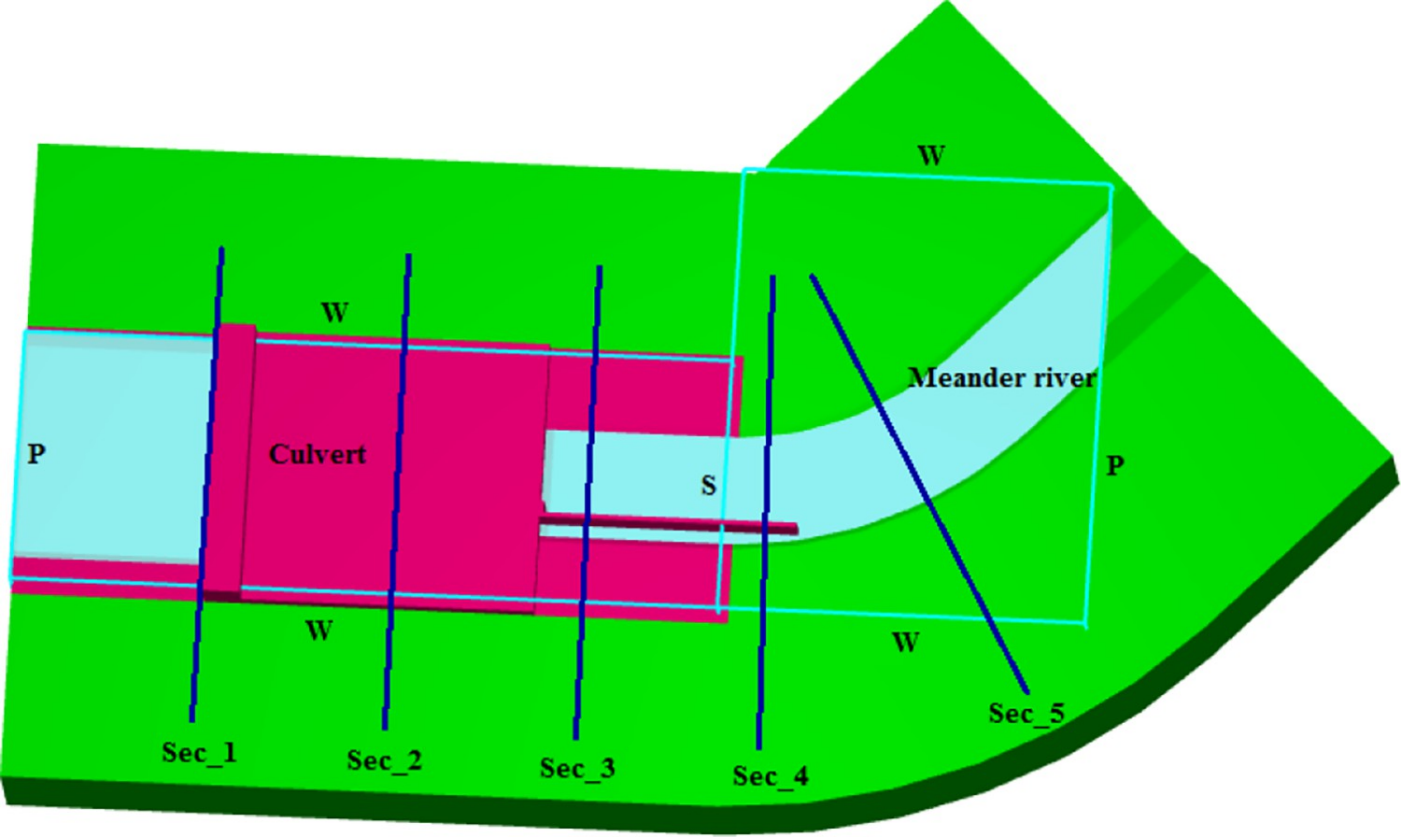
Configuration of the computational domain.

**Fig 3:**
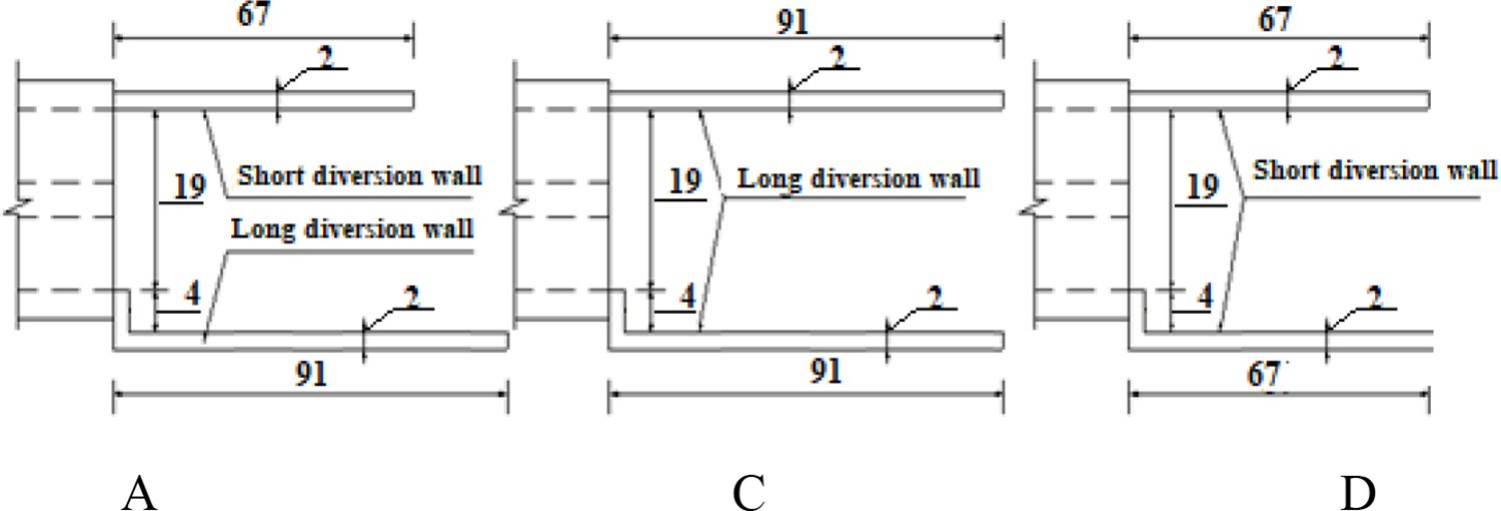
Dimension (in cm) of headwall models.

The computational domain in Flow 3D demonstrated the physical model of the Dong Nai diversion culvert, which was divided into two blocks (Fig 2): block 1 including the intake, upstream flume, and apron, was solid; and block 2 was a tail flume, which is defined by a packed sediment boundary. The upper boundary of block 1 and the lower boundary of block 2 had specific pressures corresponding to the water elevation of the three operating conditions, as shown in Table 1. All *y*-axis boundaries of blocks 1 and 2 were walls. Two objectives were set up as follows:

- Simulate hydraulic features such as flow regime, water depths, and velocities at the upstream and downstream of the intake, pressure head at the gauges on the two-box tubes of the culvert, so that the selected numerical model could be validated and the mesh sensitivity could be estimated. Only 100 seconds were required for computational time to maintain a steady flow.
- Numerically estimate the impact of alternative types of wing walls on the dimensions of scour holes after culvert, four models (A, B, C, and D) of wing walls were introduced, as seen in Table 2 and Fig 3. Model A was installed in the physical model. The other cases were scenarios established using a mathematical method. Additionally, to approach the steady flow in the whole domain of the active sediment to scour module, at least 900 s– 1000 s of computational time should be set up. This point will be explained in section 3.2.

**Table 2:** Headwall type models.

Models	Case studies
A	1 long, 1 short walls
B	No wall
C	2 long walls
D	2 short walls

Based on the criterion of non-cohesive materials after hydraulic work of TCVN 9160 combined with the magnitude of velocity at the outlet of this case study, uniform soil properties were selected (i.e., entrainment coefficient of 0.005, and mass density *δ*_*s*_ = 2650 kg/m^3^) [30]. The mean grain size (*d*_*50*_) chosen in this study was 3.6 mm.

## 3. Results and discussions

### 3.1. Validation and mesh sensitivity analysis

To validate the proposed numerical model, flow regime, water depth, and magnitude of velocity at the inlet of intake pressure heads along each culvert’s tube corresponding with the three test cases were predicted and compared with observed data. Besides, to verify mesh sensitivity, three mesh resolutions were selected to simulate hydraulic characteristics for the A model, namely 0.01 m, 0.015 m, and 0.02 m.

#### 3.1.1. Error measurement

In this study, the accuracy of the numerical model was evaluated using statistical indicators. The Normalized Root Mean Square Error (NRMSE) and the Mean Absolute Error (MAE) given in the following Eqs (6)–(7) were used to evaluate the models:

$$NRMSE=\frac{\sqrt{\frac{\sum_{i=1}^{n}{\left(X_{i,\exp}-X_{i,sim}\right)}^{2}}{n}}}{\left(X_{\exp,\max}-X_{\exp,\min}\right)}$$ (6)

$$MAE=\frac{1}{n}\sum_{i=1}^{n}\left|X_{i,\exp}-X_{i,sim}\right|$$ (7)

where *n*: number of data; *X*: hydraulic factor; *exp*: observed data; *sim*: simulated data. The lower the NRMSE and MAE values are, the better the model performance is.

#### 3.1.2 Pressure head at the bottom of boxed tubes

**Fig 4:**
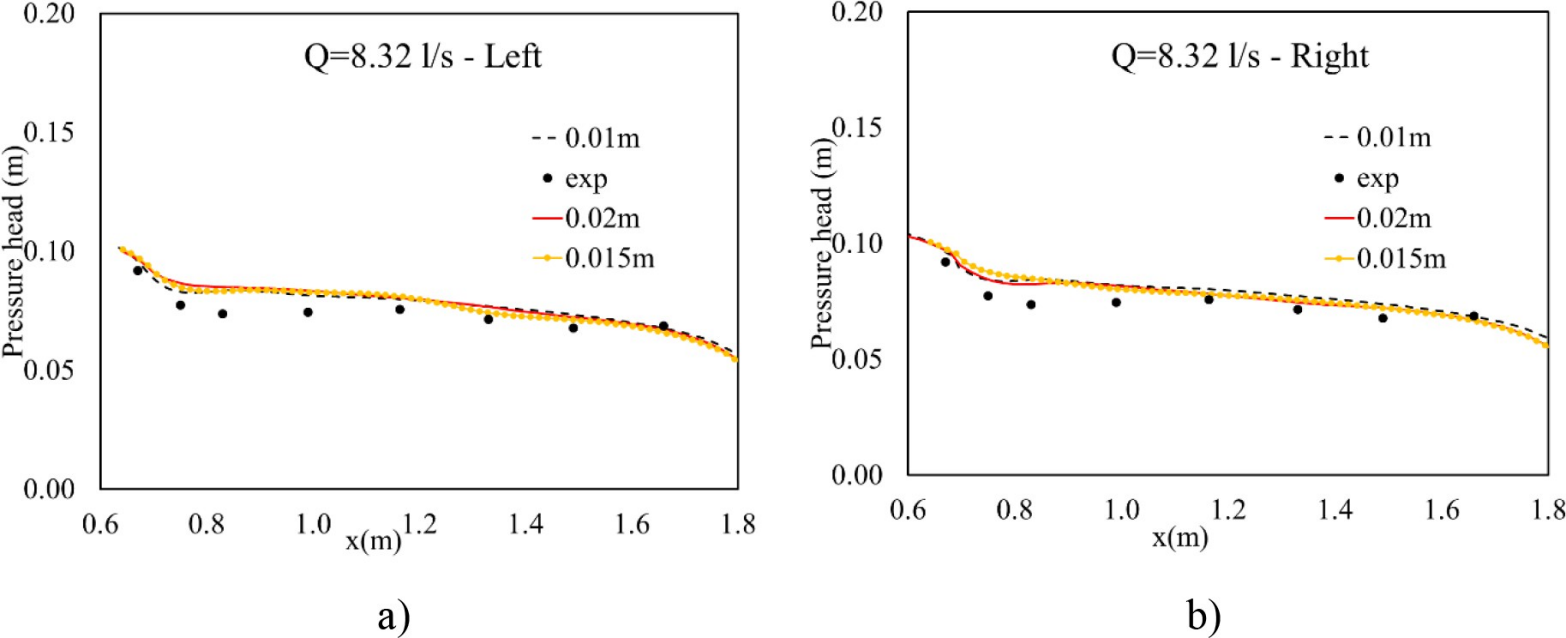
Pressure head distribution along the centerline of two tubes of Run 1. a) Left box tube; b) Right box tube.

**Fig 5:**
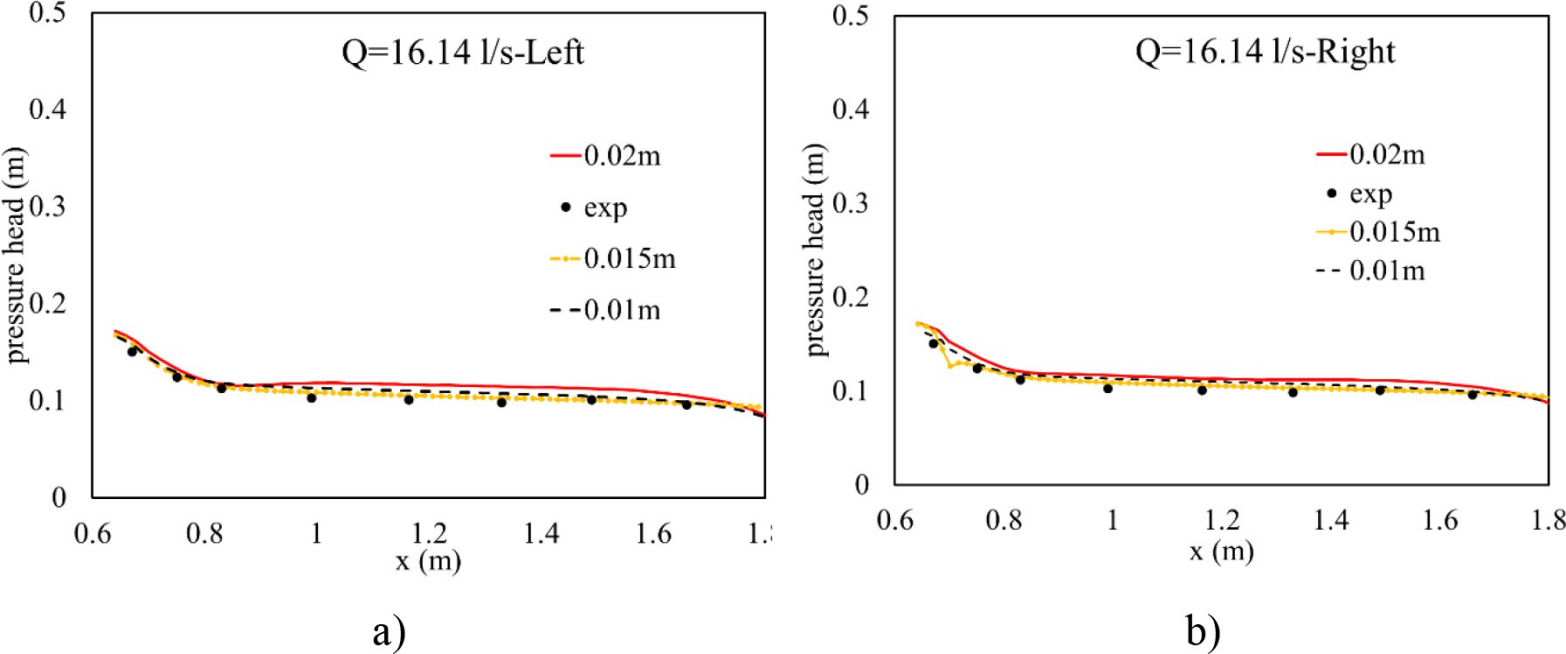
Pressure head distribution along the center line of two tubes of Run 3. a) Left box tube; b) Right box tube.

By using a set of piezometer tubes connected with eight gauge points along the central line of each tube, potential pressure heads were collected. Accounting for run 1, good matching was observed near the exit of the culvert, while overestimation between them was indicated near the inlet in Fig 4. Conversely, the much better agreement between numerical and experimental results was verified, as seen in Fig 5, when Q = 16.14 L/s. The finest mesh of 0.01 m gave the best result at the entrance of the intake tube, while the largest misplacement at the exit of the culvert was seen at this cell size. The coarsest one provided the largest difference between simulated and observed results (Fig 5) and the largest values of NRMSE and MAE in both box tubes (Table 3). Moreover, the most suitable solution was provided using a resolution mesh of 0.015 m (Fig 5), and this cell size also gave the smallest values of NRMSE and MAE, indicating the most suitable grid size (Table 3). This is because the 0.015 m grid size performed better at the culvert bottom than the others; hence, it simulated more crucial pressure at the basement of the culvert. Therefore, this cell size was taken to simulate other hydraulic features and to characterize sediment scour mechanisms at the downstream channel.

**Table 3:** NRMSE and MAE indicators of pressure head.

Q (L/s)	Indicator	Left box tube			Right box tube		
		0.01 m	0.015 m	0.02 m	0.01 m	0.015 m	0.02 m
8.32	NRMSE	0.26	0.29	0.32	0.25	0.28	0.26
16.14		0.15	0.07	0.22	0.12	0.09	0.21
8.32	MAE	0.0050	0.0056	0.0062	0.0052	0.0057	0.0070
16.14		0.0075	0.0031	0.012	0.0065	0.0026	0.0104

#### 3.1.3. Water depth and depth-averaged velocity at the entrance of the intake

**Fig 6:**
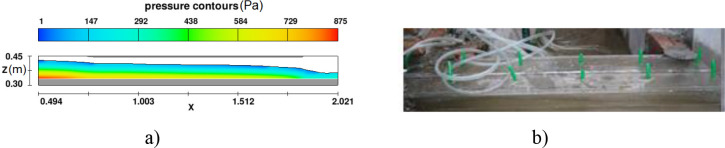
Flow regimes in a boxed culvert in Run 1. a) Numerical model; b) Physical model.

**Fig 7:**
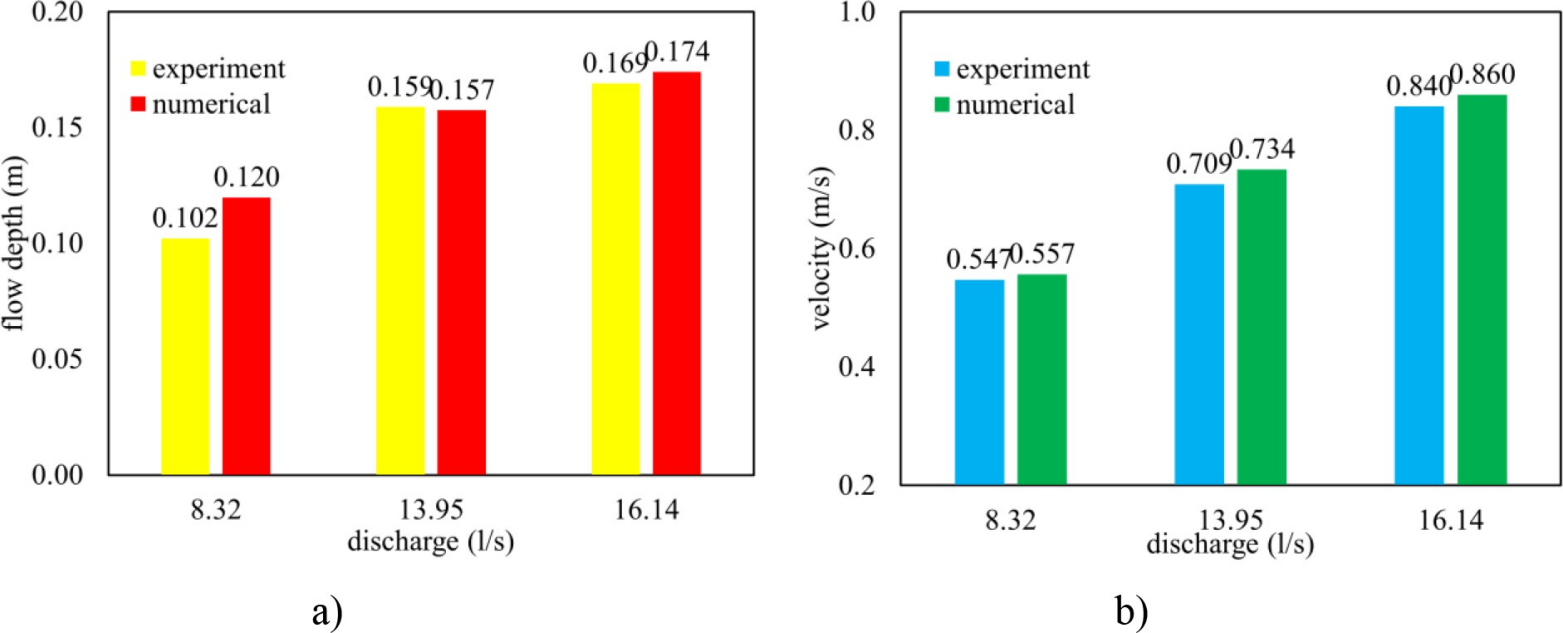
Water depth and depth-averaged velocity at section 1 in three runs. a) Flow depth; b) Depth-averaged velocity.

Both the measured data and numerical results showed that the free surface flow went through the two-box culvert in run 1 (Fig 6). Moreover, water depth and depth-averaged velocity at the entrance of intake measured in section 1 (Fig 2) showed the close agreement between observed and numerical solutions in all test cases, (Fig 7). In terms of the smallest input discharge, the relative error of water depth and velocity were 5.6% and 1.8%, respectively. For the largest one, 2.96% was estimated for the former and 2.38% for the later.

Consequently, these results indicate that Flow 3D is an efficient tool for the prediction of hydraulic characteristics.

### 3.2. Numerical simulation sediment scour in flume outlet

In this section, two modules, namely the turbulence and sediment scours of the Flow 3D package, were activated to predict scour geometry at the flume outlet. Essential computational time (*t*_*s*_) was considered as maintaining steady flow, therefore obtaining equilibrium scour depth in all case studies. In subsection 3.2.1, the influence of diversion types on the relation between time *t* and value of *d*_*s*_*/d*_*s max*_ was determined to find critical time *t*_*s*_, where *d*_*s*_ is net packed sediment changed in time, and *d*_*s max*_ is the maximum value of *d*_*s*_. This procedure was also described on [8] and [31] when investigating the numerical value of *d*_*s max*_. Then, scour behaviors after the rigid apron and at the bend segments were numerically studied.

#### 3.2.1. Computational time

Scour time is a necessary time to obtain equilibrium depth. After conducting some preliminary tests, it was observed that maximum scour depth became consistent in time when the run time reached 1000 s. Thus, we took the maximum passing discharge as a case study for this issue.

**Fig 8:**
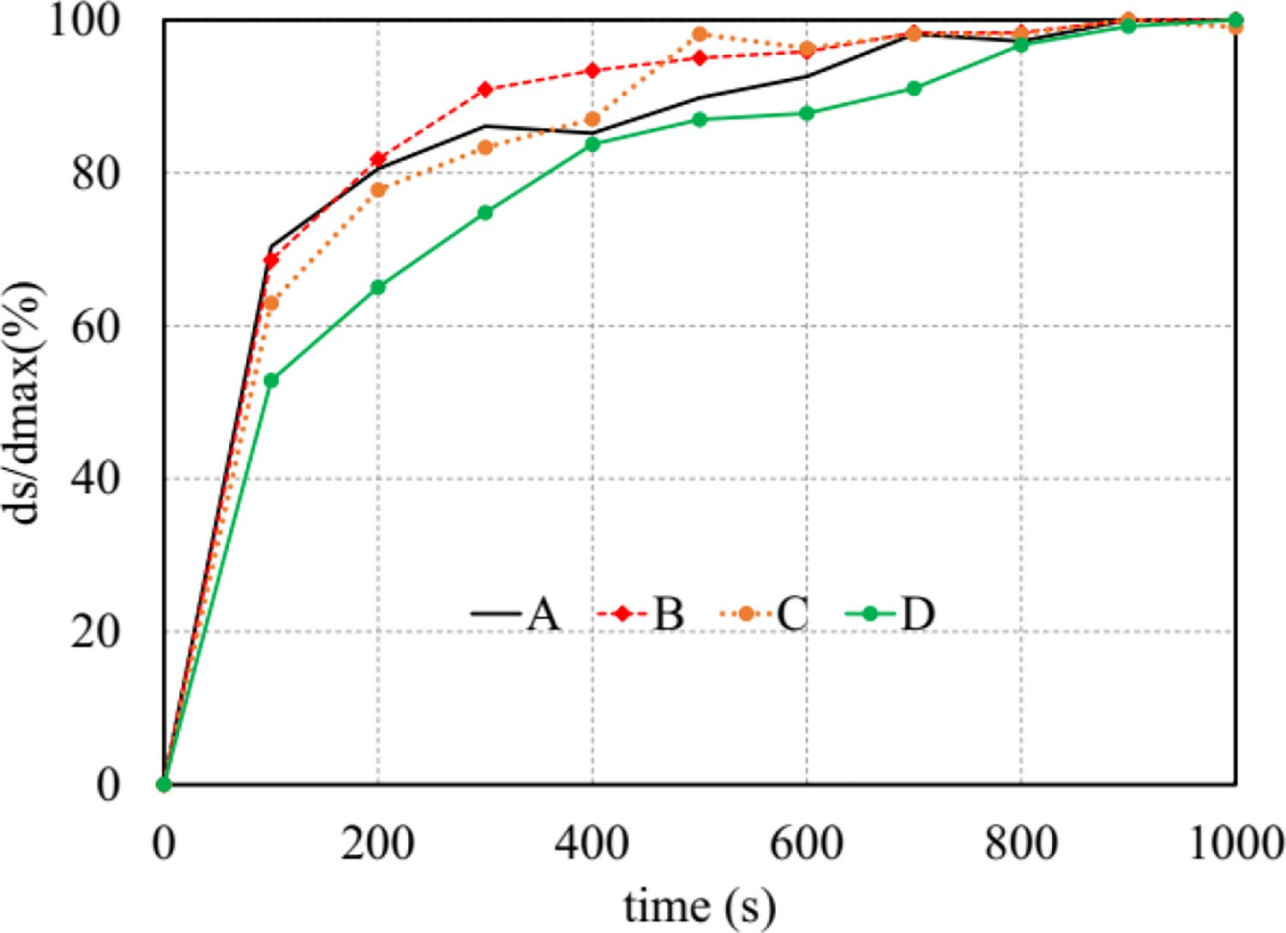
The influence of diversion walls on computational time.

In Flow 3D, maximum scour depth (*d*_*s*_) was collected every 100 seconds. The *d*_*s*_*/d*_*s max*_ ratio was used to normalize scour depth, where *d*_*s max*_ denotes the maximum scour depth. The relation between this ratio and the time corresponding to four types of wing walls was plotted in Fig 8. This figure indicated that, in the early stage, the sedimentation rate yielded by model A was greater than the others. Because, at 100 s, its *d*_*s*_*/d*_*s max*_ was 75%, while values generated by models B, C, and D were 70%, 63%, and 57%, respectively. Four types of headwalls show that all of them obtained approximately 95% of the deepest scour at 800 s (Fig 8). Consequently, 1000 s was the time required to maintain constant dimensions of scouring geometry in the packed bed of the exit channel.

Therefore, in the first scouring simulation, model A was considered as the base case to verify the influence of the roughness/*d*_*50*_ ratio (*c*_*s*_) on scour hole geometry. The results are presented in subsection 3.2.2.

#### 3.2.2 The influence of the roughness/d_50_ ratio on the deformation of flume outlet

The necessity of reduction of scours after rigid aprons has been always a crucial issue in engineering [19,24,32,33]. Soil information such as mean grain size (*d*_*50*_) and roughness are important parameters affecting the dimensions of scour hole geometry. To discover the influence of these parameters on the erosion and deposition behavior in packed downstream domains, the maximum input flow, and model A of the wing wall were selected. The morphological change of downstream flow can be divided into two zones: the area after the straight section, and the bend of the meander flume. The scour behavior of each zone can be described as follows:

- In Zone 1, the magnitude of velocity at the outlet of the culvert is much higher than that in Zone 2. The degradation of the packed sediment fume is mostly caused by the high densimetric Froude number.
- Zone 2 includes the inner and outer banks of the meander flume. Coriolis force is the main reason for deposition at the convex bank and erosion at the concave one.

**Fig 9:**
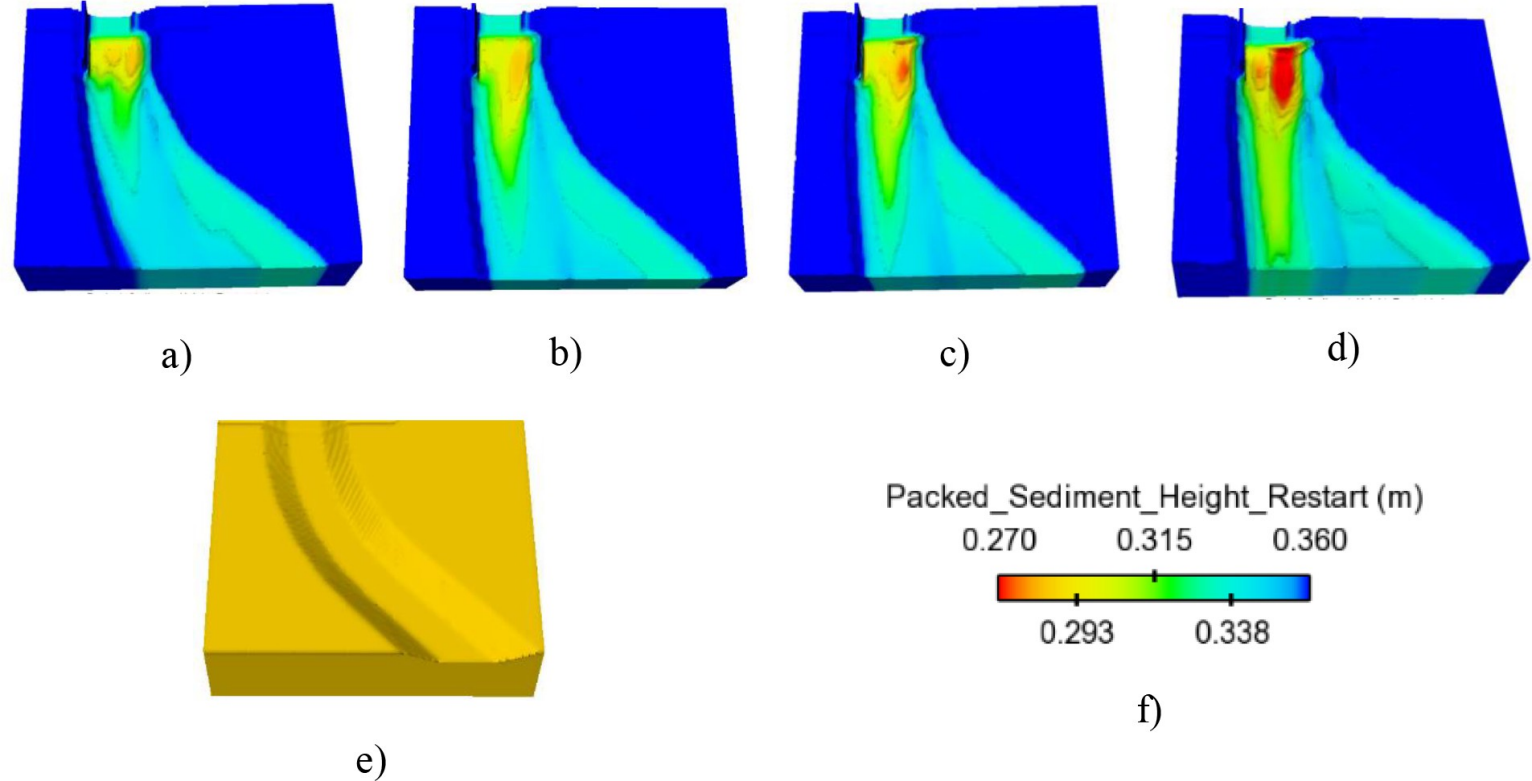
The effect of *c*_*s*_ on morphological change on the flume outlet. a) c_s_ = 1.0; b) *c*_*s*_ = 1.5; c) *c*_*s*_ = 2.0; d) *c*_*s*_ = 2.5; e) Initial fume outlet; f) Color bar of packed sediment height.

**Fig 10:**
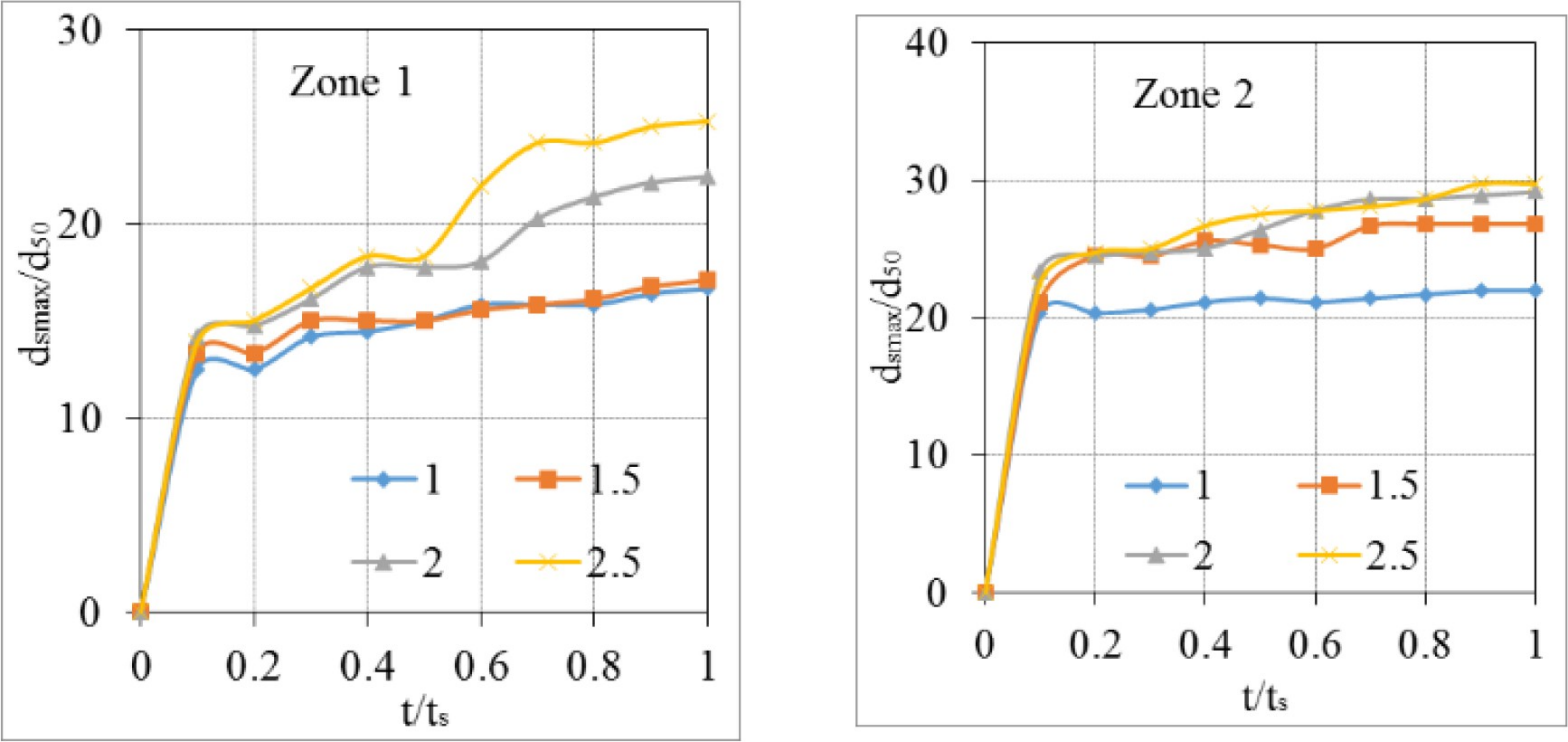
The effect of *c*_*s*_ on morphological change rate at the outlet of the culvert (Zone 1) and meander segment (Zone 2).

Accounting for various roughness/*d*_*50*_ ratios (*c*_*s*_), four values of this ratio including 1.0, 1.5, 2.0, and 2.5, were used to verify the deformation of the downstream flume, in which the suggested value of *c*_*s*_ by Flow 3D was 2.5. The development of Zone 1 caused by *c*_*s*_ = 2.5 was much larger than that yielded by a smaller value of *c*_*s*_ (Figs 9 and 10). Additionally, the degradation of two sides of the flume at Zone 2 was also significantly changed, although its shapes were quite similar in all cases. A strong erosion hole and a sand mound appeared on concave and convex banks together, respectively. The rate of *d*_*s max*_/*d*_*50*_ changes in time at Zone 2 was more consistent than that at Zone 1. The greater the value of the studied ratio was, the more deeply the local scour hole at the concave bank was obtained (Figs 9 and 10).

In comparison with the empirical results taken from [5,8], and [34], the numerical solution of *d*_*s max*_/*d*_*50*_ corresponding to four cases of *c*_*s*_ and two values of input discharge are shown in Table 4.

- (Mendoza, 1984) [5]:
$$\frac{d_{s\max}}{d_{50}}=(3.67F_{0}^{0.57}d_{50}^{-0.6}\sigma^{-0.4})D$$ (8)
- (Taha et al., 2020) [8]:
$$\frac{d_{s\max}}{d_{50}}=(0.56F_{0}+0.45\frac{h_{d}}{D}-1.05)\frac{D}{d_{50}}$$ (9)
- (Emami & Schleiss, 2010) [34]:
$$\begin{array}{l} \frac{d_{s\max}}{d_{50}}=[a\ln(F_{0})+b]\frac{D}{d_{50}}\\ a=0.6(\frac{h_{d}}{D})+1.8;\;b=1.23(\frac{h_{d}}{D})-2.25 \end{array}$$ (10)
  where:
  *F*_*0*_: Densimetric Froude; $F_{0}=u_{o}/\sqrt{(\rho_{o}/\rho -1)gd_{50}}$; *u*_*o*_ is the velocity at the exit of the culvert
  *D*: The height of the culvert
  *h*_*d*_: Water depth at downstream
  *σ*: Standard deviation of soil

**Table 4:** Relation of *c*_*s*_ and maximum scour depth ratio (*d*_*s max*_*/d*_*50*_).

Q (L/s)	h_d_/D	F_0_	Flow 3D				Empirical equations		
			*c*_*s*_ = 1.0	c_s_ = 1.5	c_s_ = 2.0	c_s_ = 2.5	Eq (8)	Eq (9)	Eq (10)
16.14	0.65	3.31	16.67	22.22	24.44	29.70	23.02	32.98	35.06
13.95	0.64	2.92	16.11	16.82	17.76	18.50	21.42	20.96	26.23

Numerical solution of maximum scour depth is highly dependent on the value of *c*_*s*_ (see Table 4). Moreover, the dimension of scour hole developed strongly when the value of *c*_*s*_ increased (see also Fig 9). Therefore, calibrating the value of *c*_*s*_ to get the reasonable numerical solution of sediment scour must be done before simulating sediment transport.

To calibrate this parameter, three empirical equations of *d*_*s max*_*/d*_*50*_ functioned of Densimetric Froude number (*F*_*0*_) were selected. Table 3 indicates that the numerical results of equilibrium scour depth taken by three values of *c*_*s*_ including 1.0, 1.5, and 2.0 are underestimated in all empirical results. While *c*_*s*_ = 2.5 gave the suitable value with analytical solutions. Therefore, this parameter was taken to numerically assess the effect of wing wall types and its dimension on morphological change in the exit meander channel.

#### 3.2.3. Effect of headwall types on erosion and deposition on channel outlet

**Fig 11:**
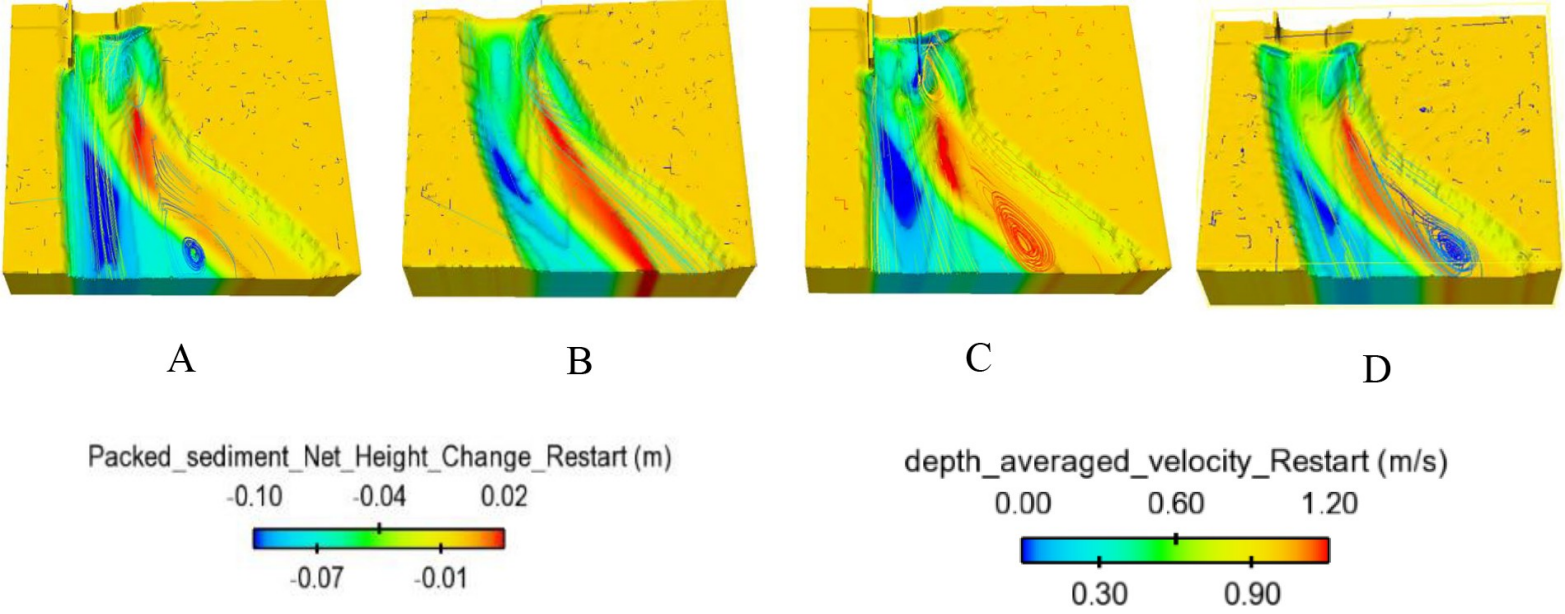
Packed sediment net change and depth-averaged velocity streamline.

**Fig 12:**
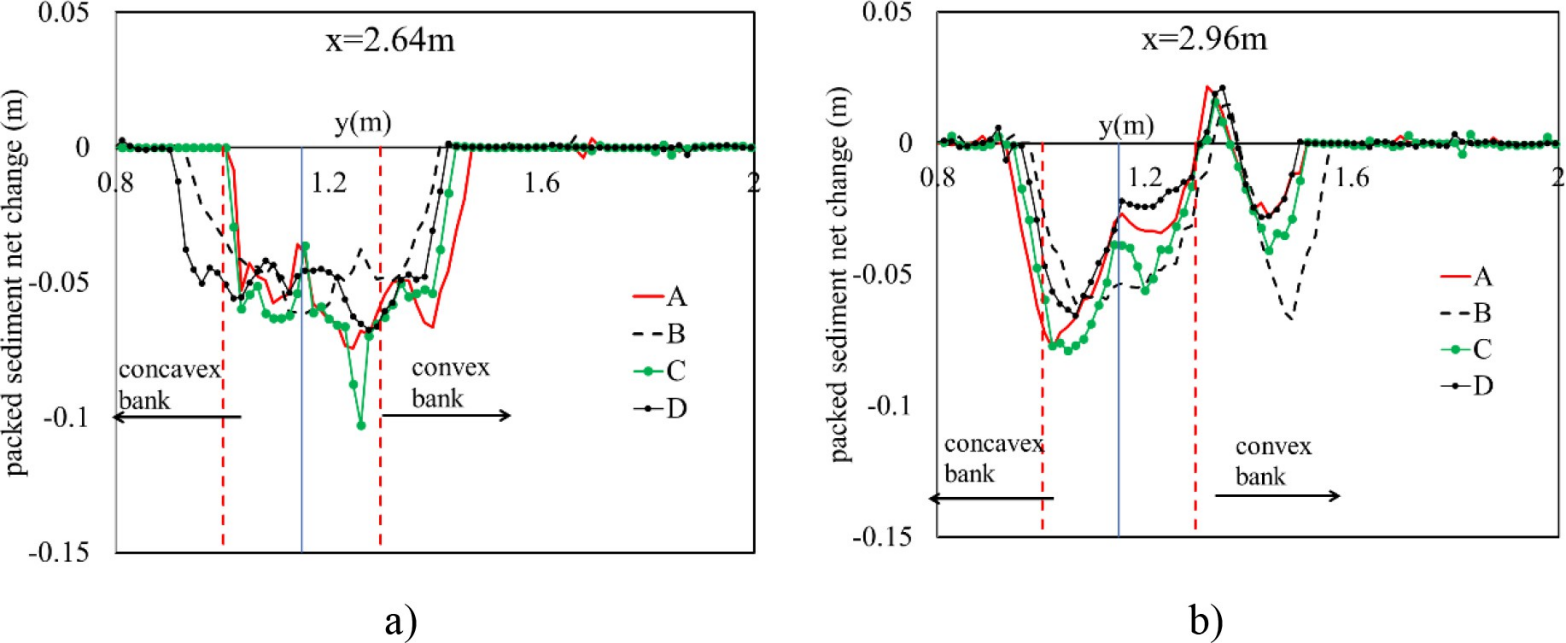
The influence of wing wall types on scour hole geometry. a) Cross section 4; b) Cross section 5.

**Fig 13:**
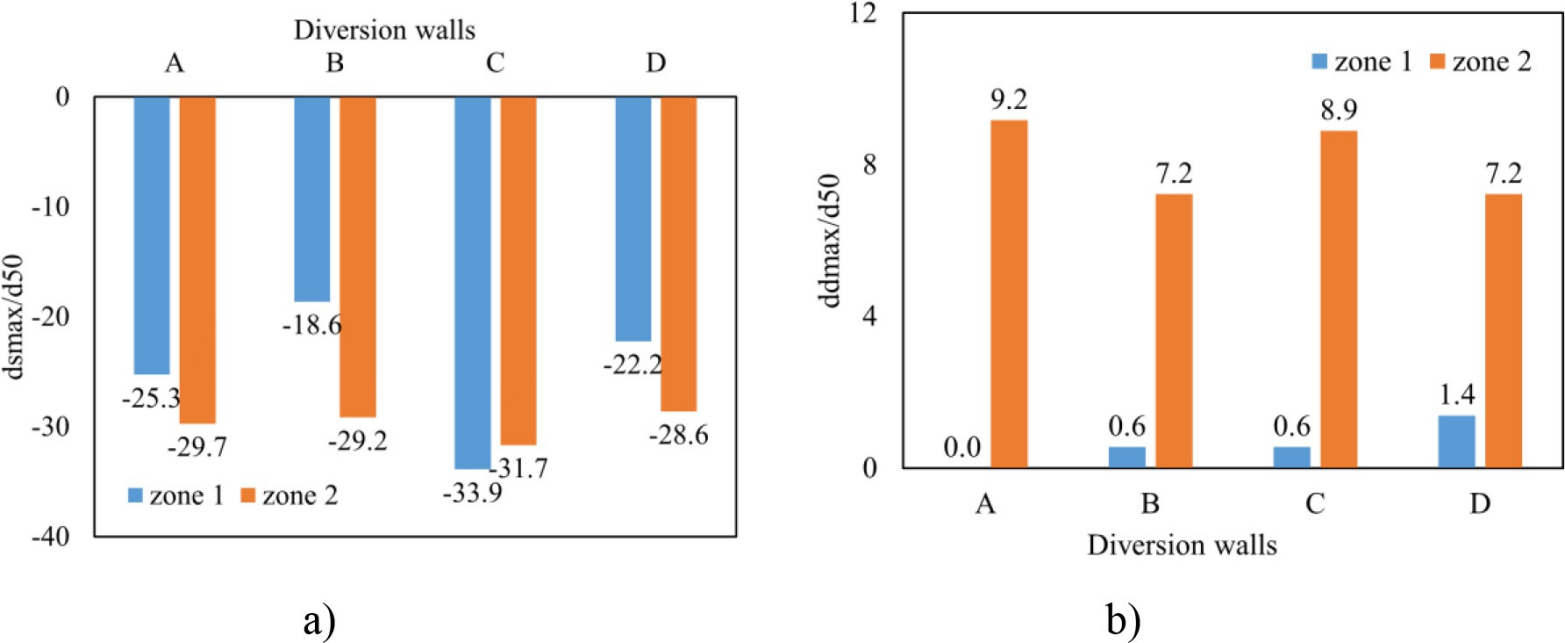
Maximum scour depth and height deposition after culvert and meander segment. a) Erosion hole; b) Deposition ridge.

**Fig 14:**
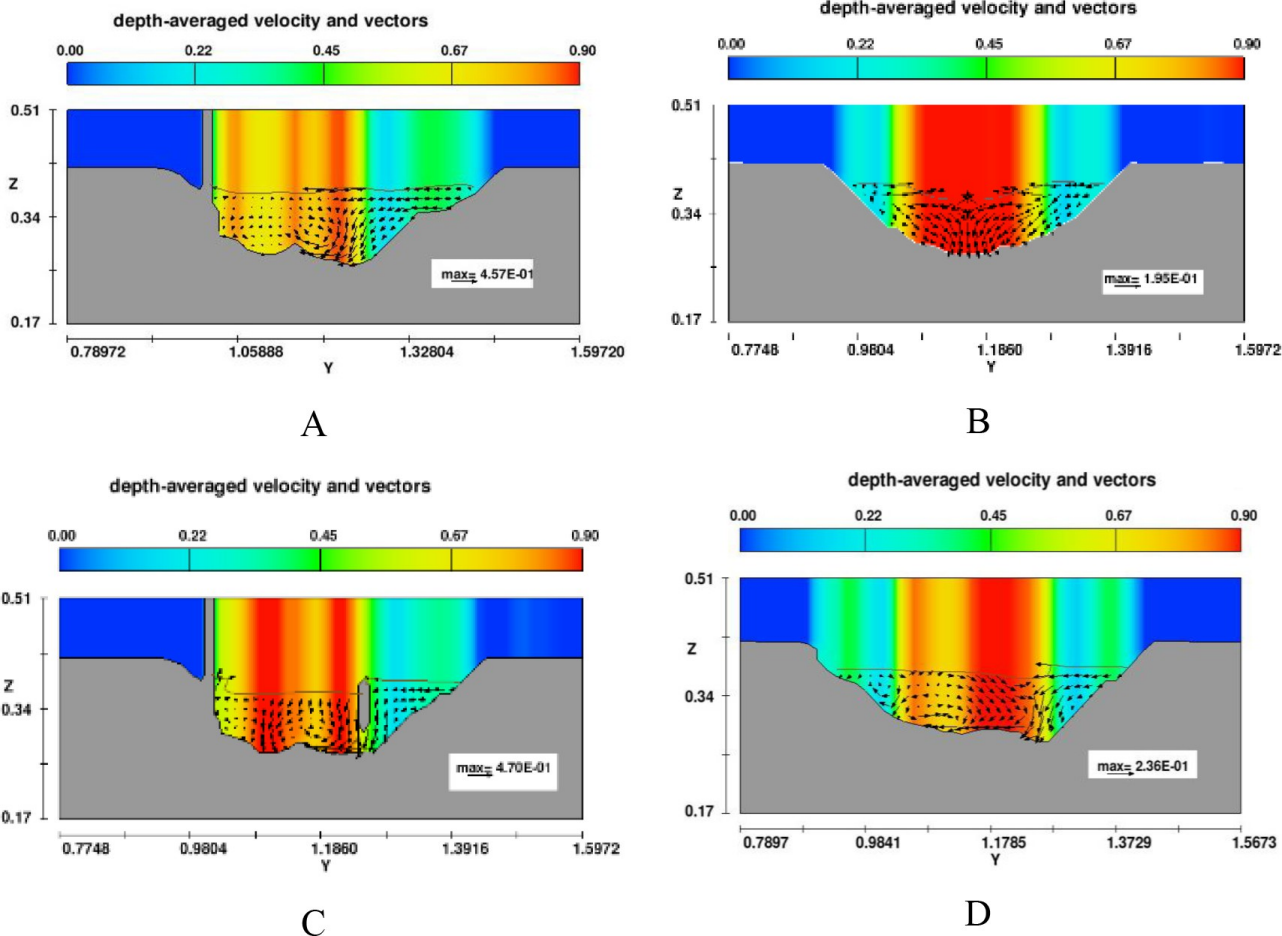
Depth-averaged velocity distributions in section 4.

**Fig 15:**
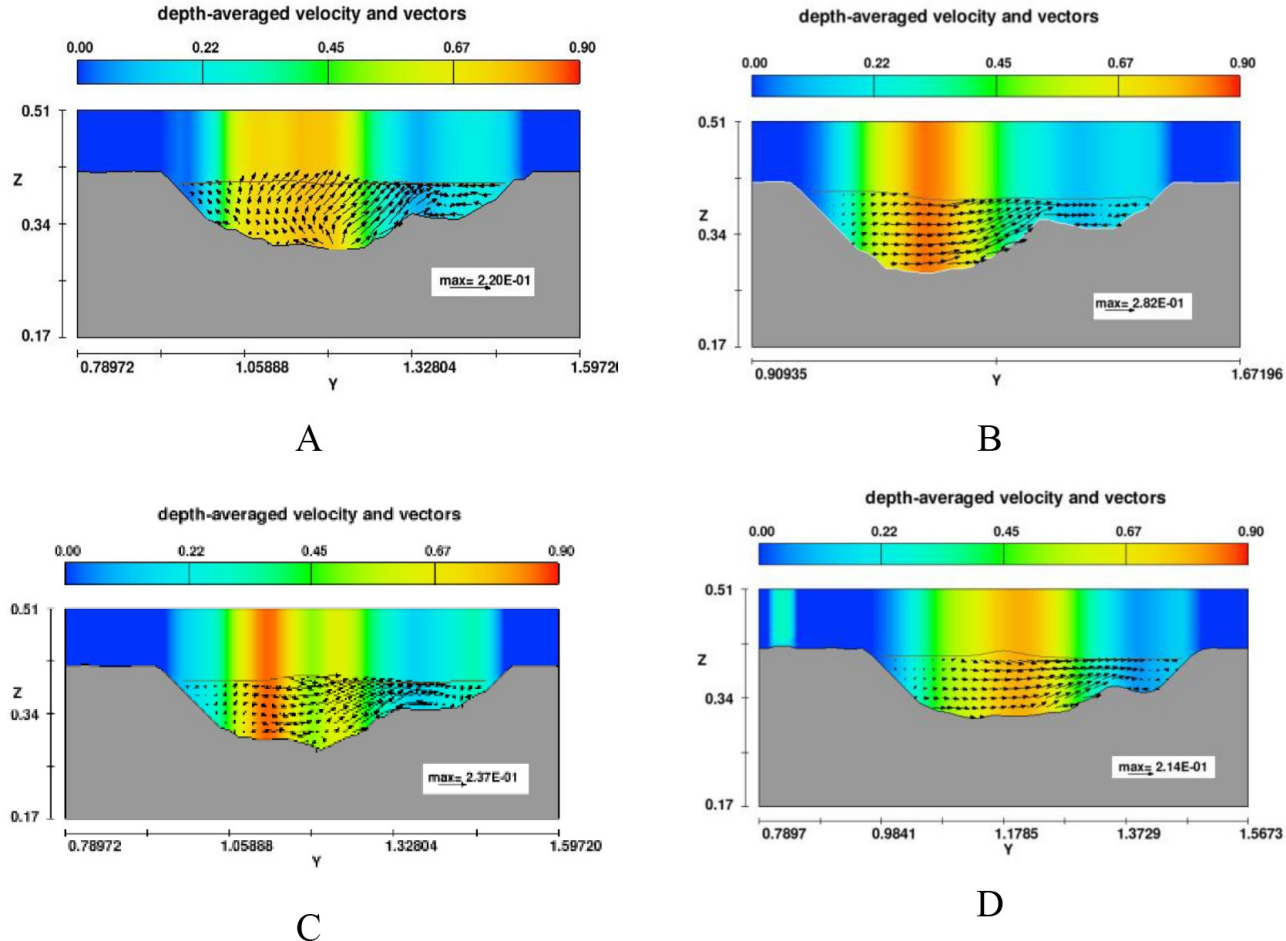
Depth-averaged velocity distributions in section 5.

In the case study, four models of diversion walls at the intake outlet were investigated for their influence on morphological changes in the two zones. The scour behavior of each zone can be described as follows:

- The scour hole was mostly developed due to high-speed velocity from the culvert at Zone 1. Numerical results showed that the slope of upstream of the scour hole was much greater than that at downstream (Fig 11). In the case of no diversion wall (model B), the geometry of the erosion hole was symmetrical, while models A, C, and D generated an asymmetrical scour hole. A higher headwall prevented erosion on the concave bank, and sedimentation developed quickly on the convex bank. With the same main grain size of the rough bed, *d*_*50*_ = 3.6 mm, the maximum scour depth was produced by model C, and its location occurred at the tip of the lower wing wall (Fig 12). The largest degraded area was also seen in model C and the smallest one was in model D (Fig 15). Particularly, Fig 12 delineated the deformation of sections 4 and 5. The long wing wall constructed on the concave bank of models A and C prevented this side from eroding. However, the deepest point occurred at Zone 1 was yielded by model C when the ratio *d*_*s max*_/*d*_*50*_ obtained a maximum value of 33.9. Zone 1 had the shallowed hole created by model B when no walls were installed (Fig 12).
- Moreover, the quantity of *d*_*s max*_/*d*_*50*_ caused by all models A, B, C, and D at Zone 2 was quite similar, i.e., 29.7, 29.2, 31.7, and 28.6, respectively (Fig 13). Cross-section 5 had the same degraded form in all models. The lower erosion hole occurred on the concave bank, and the smaller one appears on the other side. The highest sand dune was observed in model A, followed by model C, while the height of the deposition mound created by models B and D were similar (Figs 12 and 13). However, Figs 13 and 14 also illustrate that at both centers of sections 4 and 5, the long headwall (case C) created the deepest erosion in both zones.

In conclusion, accounting for the sand removal volume, two short headwalls (case D) gave the smallest area affected by deposition and sedimentation. While two long ones (case C) generated the largest area and the deepest scour depth.

### 3.3. Effect of wing wall types on hydraulic characteristics in the meander exit channel

To explain the impact of diversion walls on erosion and deposition mechanism at two zones of the exit channel, the depth-averaged velocity distributions at cross-sections 4 and 5 were investigated (see Figs 14 and 15). Model B (no headwalls) showed the maximum velocity appearing at the center of cross-section 4, which indicated why this case created the maximum scour depth at the center of this section. The material was removed from beneath the headwall on the right side of model C. Vector velocity fields also indicated vortex flow existing on the flow drained out of the culvert. On the other hand, all vector direction patterns in section 5 were from the concave side to the convex side, which means that fluid and soil particles may transport from the left bank to the right bank. Moreover, the streamline of depth-averaged velocity displayed in the 3D view also indicated that vortex flow appeared near the tip of the wing wall on the convex bank in all cases (Fig 15).

**Fig 16:**
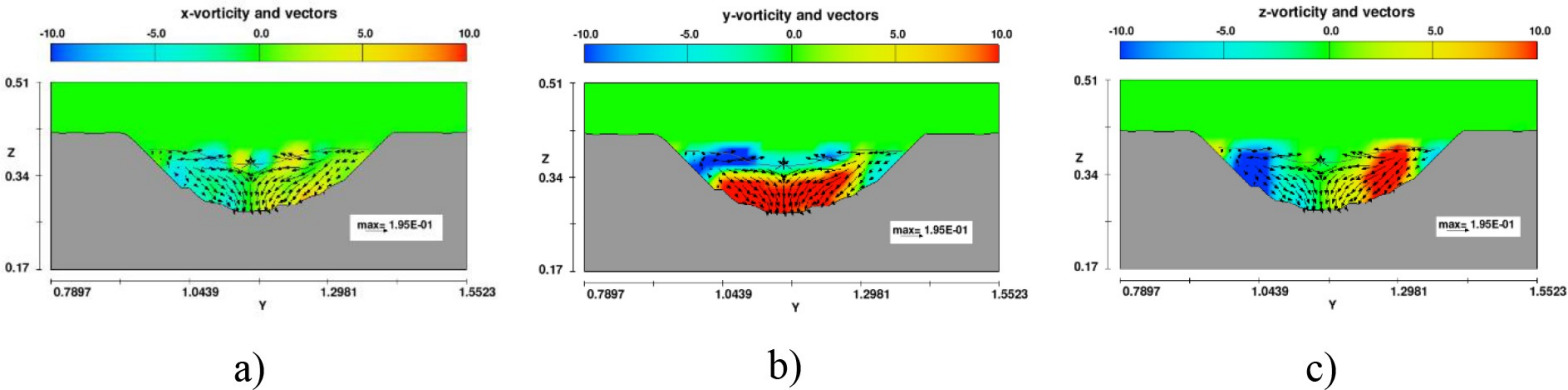
Distribution of vortex components distribution at section 4 in model B. a) *x*-vorticity; b) *y*-vorticity; c) *z*-vorticity.

**Fig 17:**
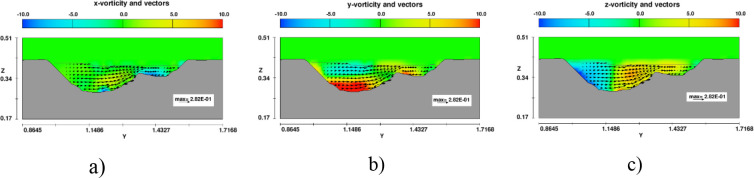
Distribution of vortex components distribution at section 5 in model B. a) *x*-vorticity; b) *y*-vorticity; c) *z*-vorticity.

At the bent segment, the bed topography was strongly three-dimensional deformed (see Fig 9) [35]. The pools always formed near the outer banks, whereas the deposition fronts always formed near the inner bank. Two driving forces accounting for the gravity force and the Coriolis force are reasons of whirlpool occurred at the meander segment. Figs 16 and 17 presented the distribution of three vortex components in sections 4 and 5 in case of no headwalls (case B). At cross-section 4, all vortex components were distributed symmetrically because it was not influenced by the curved flow (Fig 16). However, in section 5, in the *y*-direction, clockwise and counterclockwise flows incorporated with positive and negative values of vorticity led to a whirling motion of the fluid. The higher the magnitude of the vorticity value was, the stronger whirlpool occurred (Fig 17). Hence, the soil element was detached and moved as bed-load or suspended sediment along the main flow direction and from the concave bank to the convex bank.

## 4. Conclusion

The 3D CFD model has been used widely in many complicated hydrodynamic problems. Two modules, namely turbulence and sediment scour, were selected to simulate several hydraulic characteristics of flow over a culvert coupling with a packed bed of exit flume. The numerical results of water depth, velocity and pressure head, and flow regime were compared with observed data, showing good agreement between the three operating conditions. Mesh sensitivity was analyzed with three mesh resolution sizes and indicated that a grid size of 0.015 m was adequate in simulating several sophisticated hydraulic features.

The study showed that the critical time of 1000 s was necessary to maintain a steady state for all simulation cases accounting for sediment scour phenomena. With four models of diversion walls at the outlet of the intake as well as various roughness/*d*_*50*_ ratios, the influence of these scenarios on sedimentation and deposition after the straight and meander segments were discovered. The conclusions were as follows:

- Two distinct zones were formed in the flume outlet. The deeper zone was formed due to high-speed velocity after the rigid apron. The erosion on the concave bank and deposition on the convex bank occurred at the meander segment due to the effects of the meandering.
- The bed deformation was highly sensitive with roughness/*d*_*50*_ ratio (*c*_*s*_). Particularly, the higher this ratio was, the deeper and larger the volume of bed deformation was. In comparison with empirical solutions, the numerical result of maximum scour depth yielded by value *c*_*s*_ of 2.5 was the most reasonable.
- The influence of the four models of the wing wall on morphological change at the flume outlet was investigated. Two short headwalls caused the smallest erosion depth in Zone 1, and two long walls generated the deepest erosion hole in this area. A long wingwall on the concave bank of models A and C created the largest deformation area in this bank. Four models also had an approximately equal height of sand mounds at the convex bank. In terms of the smallest volume of bed change, two short headwalls were the best solution.
